# Unusual Midline Chest Wall Cellulitis in an Infant Without Trauma or Skin Barrier Disruption

**DOI:** 10.7759/cureus.94633

**Published:** 2025-10-15

**Authors:** Morihide Kanawa, Hiro Nakao, Tatsuki Ikuse, Mitsuru Kubota, Kensuke Shoji

**Affiliations:** 1 Department of Postgraduate Education and Training, National Center for Child Health and Development, Tokyo, JPN; 2 Department of General Pediatrics, National Center for Child Health and Development, Tokyo, JPN; 3 Department of Infectious Diseases, National Center for Child Health and Development, Tokyo, JPN

**Keywords:** cefazolin, hematogenous spread, mssa bacteremia, pediatric cellulitis, thoracic wall

## Abstract

Cellulitis in children usually develops on the extremities or face following minor trauma or skin barrier disruption, and involvement of the trunk is uncommon. We report a one-year-old previously healthy boy who presented with fever and a rapidly enlarging anterior chest wall mass. Laboratory tests revealed elevated inflammatory markers without evidence of immunological abnormality. Ultrasonography showed a 12-mm swelling beneath the lower sternum with increased blood flow but no abscess. Cellulitis was diagnosed, and intravenous cefazolin was initiated. Blood culture obtained on admission grew methicillin-susceptible *Staphylococcus aureus*. On hospital day nine, contrast-enhanced magnetic resonance imaging demonstrated cellulitis localized to the xiphoid process without evidence of osteomyelitis. The patient completed two weeks of intravenous cefazolin and was discharged without complications. In this case, the absence of trauma, skin barrier disruption, or underlying disease raises the possibility of hematogenous dissemination of *S. aureus* as the cause of infection. Although rare, hematogenous cellulitis should be considered in pediatric patients presenting with soft-tissue infection at unusual sites. Imaging and microbiological evaluation may be helpful in atypical cases to support diagnosis and guide management.

## Introduction

In children, cellulitis most frequently affects the extremities or facial areas, often following minor trauma or skin disruption [1]. In contrast, cellulitis of the trunk is rare. In a cohort comprising both adults and children, cellulitis of the trunk accounted for only 4.5% of all cases, underscoring its rarity [2]. When evaluating an acquired anterior chest wall mass in children, the differential diagnosis includes trauma, benign tumor, malignant neoplasm, and inflammatory or infectious causes such as Tietze syndrome, cyst, sternal osteomyelitis, and abscess. Although cellulitis is generally diagnosed clinically, imaging modalities, such as ultrasonography and magnetic resonance imaging (MRI), may assist in challenging cases, that is, atypical or complicated cases, by ruling out abscess and by evaluating the extent of infection, including possible deep involvement, the risk of systemic spread, or overlap with other chest wall conditions.

## Case presentation

**Figure 1:**
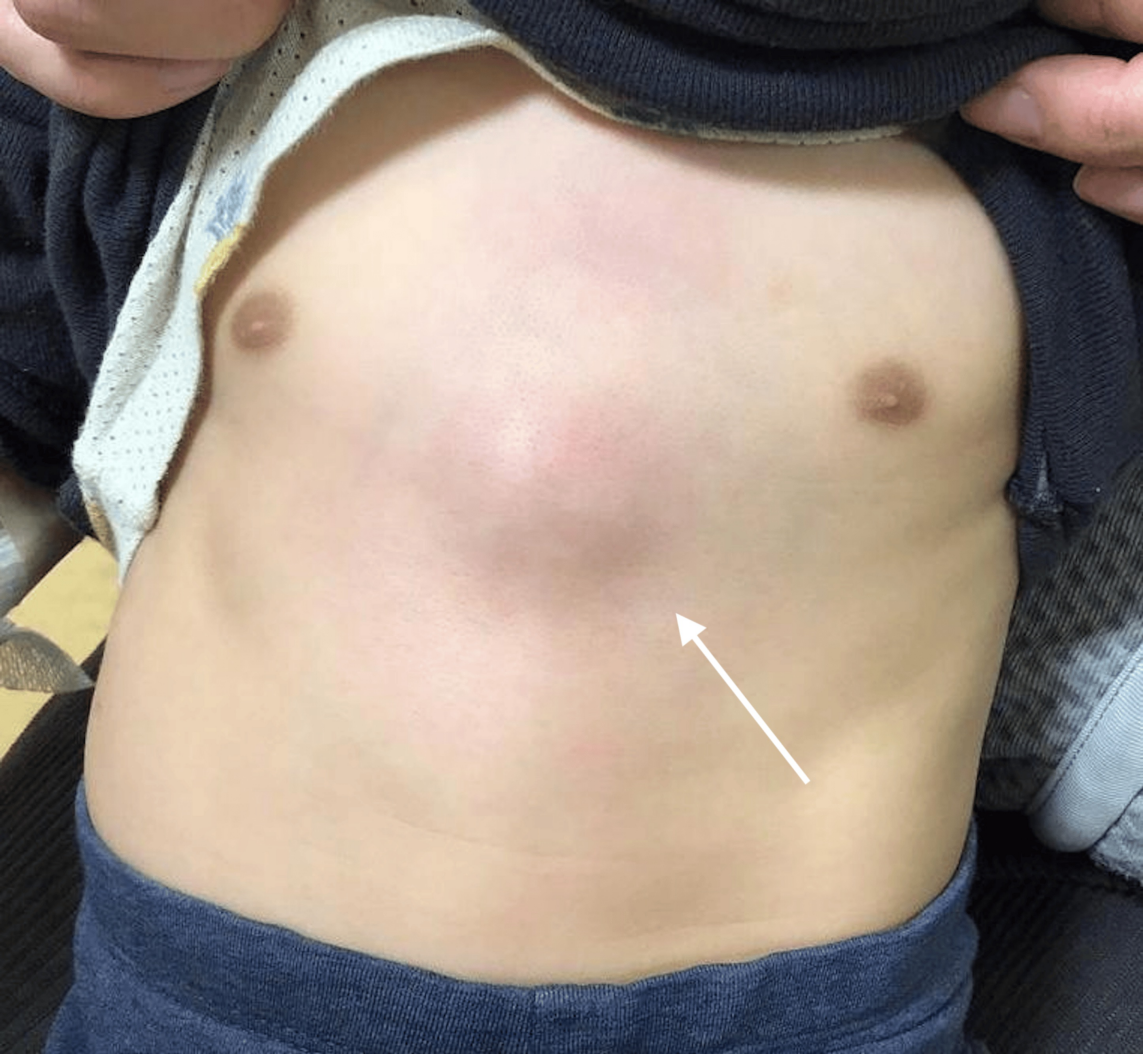
Photograph of the anterior chest wall showing a well-circumscribed, smooth-marginated, 30 mm subcutaneous mass on the midline over the xiphoid process.

A one-year-old boy was referred to our hospital for evaluation of a mass in the anterior chest. He had had a fever starting four days before admission, and the parent reported noticing the swelling a day before. The patient was up to date with routine immunizations, including Bacillus Calmette-Guerin (BCG). He had no notable perinatal, developmental, or past medical history. He had no trauma. On presentation, he was afebrile, with a pulse of 110 beats/min and a respiratory rate of 36 breaths/min. The patient’s weight was 8.4 kg, height was 76 cm, and the calculated Kaup index was 14.5. Physical examination revealed a well-circumscribed, smooth-marginated, protruding lesion measuring 30 mm in diameter on the anterior chest, surrounded by an area of pale erythema measuring 50 mm in diameter, which had no tenderness, heat, or mobility (Figure 1).

No cardiac murmur was detected. Echocardiography was not performed, as there were no clinical signs suggestive of endocarditis. The results of laboratory testing are shown in Table 1.

**Table 1:** Laboratory data Unmarked values obtained on hospital day 1, †day 3, and ‡day 8 HBs: Hepatitis B surface.

Parameter	Patient value	Reference value
White blood cells	10460/μL	4300–19600
Neutrophils	40.9%	20–35
Lymphocytes	48.1%	55–70
Monocytes	9.9%	2–8
Eosinophils	0.8%	1–4
Basophils	0.3%	0–1
Red blood cells	405×10^4^/μL	393–538
Hemoglobin	10.2 g/dL	10.5–14.1
Platelets	39.6×10^4^/μL	16.8–65.0
C3^‡^	105 mg/dL	65–135
C4^‡^	21 mg/dL	17–45
CH50^‡^	40.7 CH50/mL	25.0–48.0
IgA^‡^	57 mg/dL	16–128
IgE^‡^	538.0 IU/mL	5.4–1100.0
IgG^‡^	650 mg/dL	460–1220
IgM^‡^	120 mg/dL	57–260
HBs antibody^‡^	Positive	–
Total bilirubin	0.31 mg/dL	0.16–0.67
Aspartate aminotransferase	33 U/L	23–57
Alanine aminotransferase	19 U/L	9–38
Lactate dehydrogenase	285 U/L	202–437
Total protein	6.8 g/dL	5.7–7.5
Albumin	3.9 g/dL	3.4–4.7
Blood urea nitrogen	9.4 mg/dL	3.7–18.6
Creatinine	0.18 mg/dL	0.16–0.33
Sodium	134 mmol/L	135–143
Chloride	102 mmol/L	101–110
Potassium	4.8 mmol/L	3.6–5.1
Creatine kinase	97 U/L	39–299
C-reactive protein	5.09 mg/dL	<0.3
Soluble interleukin-2 receptor^†^	829 U/mL	157–474
Erythrocyte sedimentation rate^‡^	19 mm/h	0–10

**Figure 2:**
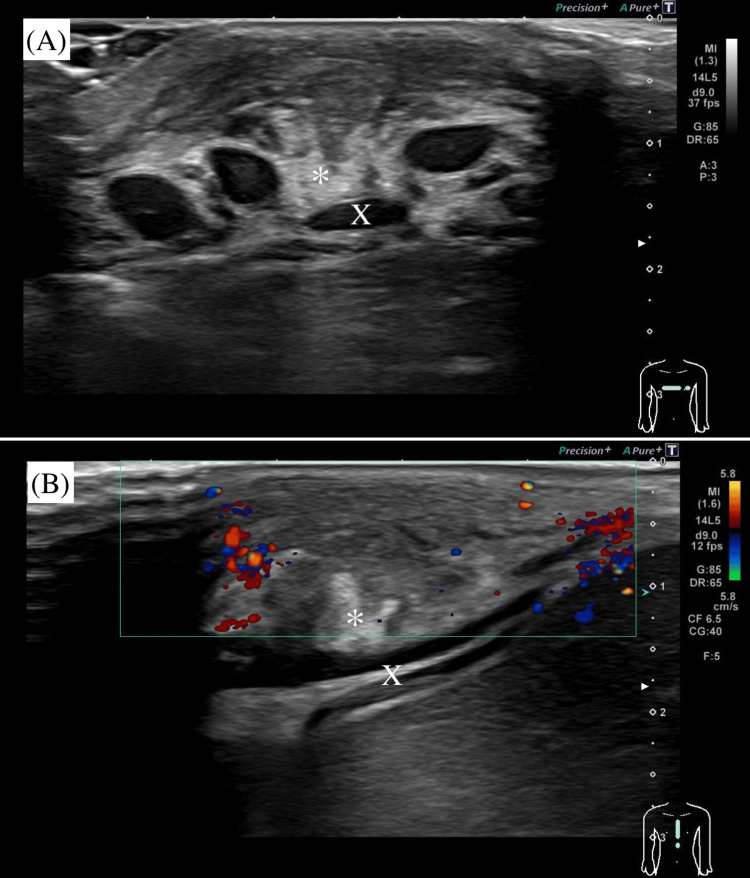
Ultrasonographic image of the anterior chest wall showing a 12-mm subcutaneous hypoechoic lesion (*) with increased peripheral vascularity, located anterior to the xiphoid process (X). No evidence of abscess was observed.

Inflammatory markers were elevated, whereas immunological test results were within normal range. Total IgE was elevated at 538 IU/mL. However, the patient had no history or clinical features suggestive of atopy, such as eczema, wheezing, or allergic rhinitis, and no signs consistent with hyper-IgE syndrome. Soluble interleukin-2 receptor was measured as part of the evaluation for possible hematological disorders. Ultrasonography revealed a 12-mm swelling in soft tissue located anterior to the lower end of the sternum, corresponding to the xiphoid process and increasing blood flow around the mass, with no evidence of abscess (Figure 2).

**Figure 3:**
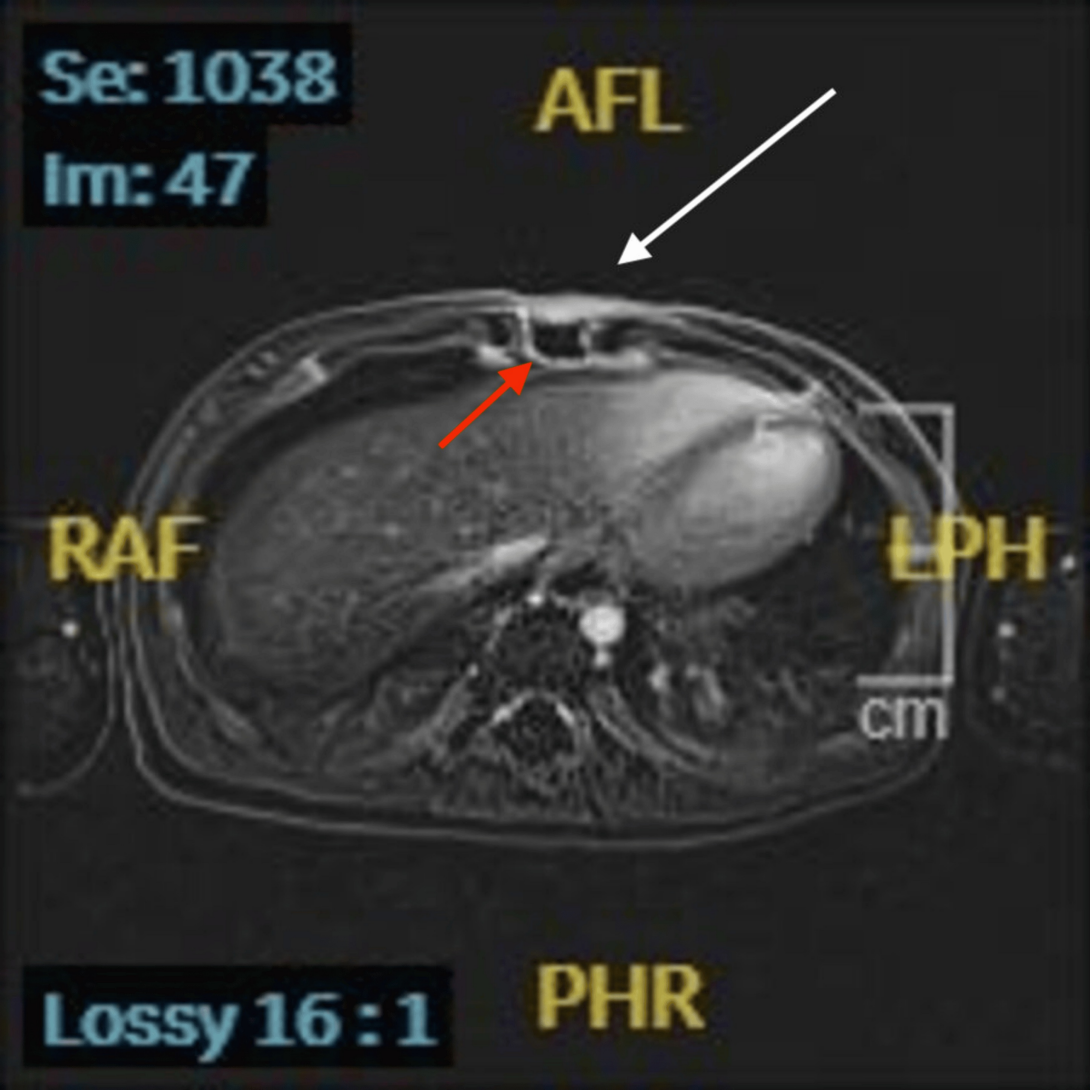
Contrast-enhanced axial T1-weighted MRI demonstrating subcutaneous swelling and enhancement (white arrow) at the xiphoid level (red arrow), consistent with cellulitis.

Cellulitis was diagnosed, and intravenous cefazolin (100 mg/kg/day, every 8 hours) was administered. Blood culture on admission was positive for methicillin-susceptible *Staphylococcus aureus* the next day. Follow-up blood cultures obtained on hospital day three (two sets) were both negative. During hospitalization, the patient remained afebrile. The erythema resolved by hospital day three, and the swelling gradually subsided, disappearing completely by hospital day eight. On hospital day nine, contrast-enhanced MRI demonstrated inflammatory changes confined to the soft tissue overlying the xiphoid process, without evidence of osteomyelitis (Figure 3). 

The patient completed two weeks of cefazolin and was discharged uneventfully.

## Discussion

In this patient, the lesion occurred on the anterior chest wall, a highly unusual site for cellulitis. More importantly, however, the clinical presentation was atypical: the lesion was painless, without warmth, and the patient remained afebrile at admission. This lack of classical inflammatory signs contributed to the diagnostic challenge. Additionally, the absence of skin barrier disruption or underlying chronic condition made the source of infection unclear. BCG vaccination has been reported as a potential cause of soft tissue infection in the chest wall [3]. In the present case, testing for mycobacteria, including BCG, was not performed. However, since *S. aureus* was isolated from blood cultures and the patient improved with antibiotic therapy targeting *S. aureus*, mycobacteria were not suspected as the causative pathogen. Several reports have described infections around the sternum caused by a congenital dermoid sinus of the anterior chest region [4]; however, in our case, ultrasonography demonstrated no fistulous tract, making this diagnosis unlikely. 

Although the clinical presentation in our case was atypical, several findings supported the diagnosis of cellulitis. Laboratory testing showed elevated inflammatory markers, blood culture yielded *S. aureus*, and imaging revealed inflammatory changes limited to the subcutaneous tissue without abscess formation. Moreover, the lesion improved promptly with intravenous cefazolin. Taken together, these findings confirmed the diagnosis of cellulitis despite the unusual clinical appearance. In pediatric skin and soft-tissue infections, ultrasonography has been reported to have a sensitivity of 89.9% and specificity of 79.9% for distinguishing cellulitis from abscess [5], supporting its role as a useful adjunct while acknowledging its limitations. Moreover, cellulitis and osteoarticular infection have been reported to coexist in 11% of pediatric cases [6], and MRI is regarded as superior for evaluating possible osteomyelitis or deep extension. In our case, ultrasonography helped characterize the lesion, and MRI appropriately excluded osteomyelitis.

In our case, immunoglobulin levels, complement components, and anti-HBs titers were all within normal limits, suggesting no apparent immunodeficiency. However, we acknowledge that this work-up does not exclude all forms of primary immunodeficiency. Disorders such as chronic granulomatous disease or hyper-IgE syndrome, which can predispose patients to *S. aureus* infection, may not be ruled out by these tests alone. 

Infective endocarditis was considered as a differential diagnosis in view of the history of fever and positive blood culture. However, the patient had no cardiac murmur or other findings suggestive of infective endocarditis, and the chest wall lesion was consistent with localized cellulitis rather than a secondary embolic lesion. In addition, follow-up blood cultures obtained on hospital day three were negative, arguing against persistent bacteremia, which is typically required for the diagnosis of endocarditis. Therefore, echocardiography was not performed, and endocarditis was considered unlikely.

In this case, we considered the possibility that bacteremia originated from an unrecognized primary focus and subsequently localized to the chest wall. Alternatively, cellulitis itself may have represented the primary source of bacteremia, as *S. aureus* skin infections are a well-known cause of bloodstream infection. However, the atypical clinical presentation at admission and the absence of local inflammatory signs made it difficult to determine whether the chest wall lesion was the source or a metastatic focus. 

Hematogenous dissemination of *S. aureus* to the skin is rarely documented. Cutaneous lesions in bacteremia are usually observed in the setting of metastatic infection or infective endocarditis rather than as isolated cellulitis. Del Giudice et al. noted that skin manifestations of *S. aureus* bacteremia without endocarditis are extremely rare [7]. In children, reports of true hematogenous cellulitis remain scarce. 

Dissemination is facilitated by microbial virulence factors such as α-toxin, protein A, and surface adhesins (MSCRAMMs), including clumping factor and fibronectin-binding proteins. Some strains harbor Panton-Valentine leukocidin (PVL), which is strongly associated with recurrent or deep abscesses; although its contribution to invasive dissemination is variable, both PVL and α-toxin can intensify local inflammation [8]. Host defenses, particularly intact skin barriers, neutrophil function, and innate immunity, are essential to prevent spread, while inborn errors of immunity may predispose certain patients to invasive or recurrent *S. aureus* infection [9]. Although the available evidence is limited, the rarity of pediatric cases highlights the value of individual reports. In our patient, the presence of *S. aureus* bacteremia without features of endocarditis, imaging evidence of localized cellulitis without abscess or osteomyelitis, and resolution under pathogen-directed therapy support hematogenous cellulitis as a plausible explanation, while acknowledging both its rarity in children and the alternative possibility that the chest wall lesion represented the primary focus of bacteremia.

A major limitation of this report is that histopathological confirmation was not obtained, as a skin biopsy was not performed. Therefore, the precise etiology of the lesion cannot be definitively established. While hematogenous dissemination of *S. aureus* was considered most plausible, other possibilities include rupture of a congenital cyst with secondary infection, scrofuloderma arising from an underlying tuberculous focus, or secondary infection following an insect bite. Recognition of these alternatives highlights the diagnostic uncertainty and the importance of considering a broad differential diagnosis when evaluating midline chest wall lesions in children.

Notably, the patient presented with fever four days before admission, and a positive blood culture for methicillin-susceptible *S. aureus* was obtained. These findings raise the possibility of hematogenous dissemination, whereby bacteremia from an unrecognized primary focus resulted in localization to the anterior chest wall.

Furthermore, this case underscores the clinical significance of midline inflammatory lesions in children. Such presentations may represent the initial manifestation of systemic infection or serve as a portal for dissemination. Prompt recognition, microbiological evaluation, and appropriate therapy are essential to prevent progression to severe complications.

## Conclusions

Although hematogenous cellulitis is rare, particularly in the absence of underlying immunodeficiency or apparent skin barrier disruption, this case highlights that it should be considered in children presenting with soft-tissue infection at uncommon sites. Recognition of such cases is important, as delayed diagnosis may lead to progression and more severe complications. When the presentation is atypical or the site is unusual, imaging and microbiological evaluation may be considered as part of the differential diagnosis to guide management. Further accumulation of similar reports will help to clarify the clinical characteristics and optimal management of hematogenous cellulitis in pediatric patients, and to raise awareness that midline inflammatory lesions in children may herald systemic infection and therefore warrant careful evaluation.
